# Salivary markers of oxidative stress and periodontal pathogens in patients with periodontitis from Santander, Colombia

**DOI:** 10.7705/biomedica.5149

**Published:** 2020-08-20

**Authors:** Juana P. Sánchez-Villamil, Carolina Pino-Vélez, Juanita Trejos-Suárez, Néstor Cardona, Ana Lucía España, Pedro A. Alfonso

**Affiliations:** 1 Facultad de Odontología, Universidad Antonio Nariño, Bucaramanga, Colombia Universidad Antonio Nariño Facultad de Odontología Universidad Antonio Nariño Bucaramanga Colombia; 2 Grupo de Investigación Biomédica Traslacional, Centro de Investigaciones, Fundación Cardiovascular de Colombia, Santander, Colombia Grupo de Investigación Biomédica Traslacional Fundación Cardiovascular de Colombia Santander Colombia; 3 Especialización en Periodoncia, Facultad de Odontología, Universidad Santo Tomás, Bucaramanga, Colombia Universidad Santo Tomás Facultad de Odontología Universidad Santo Tomás Bucaramanga Colombia; 4 Grupo de Investigación en Manejo Clínico, Facultad de Ciencias de la Salud, Universidad de Santander, Bucaramanga, Colombia Universidad de Santander Facultad de Ciencias de la Salud Universidad de Santander Bucaramanga Colombia; 5 Facultad de Odontología, Universidad Antonio Nariño, Armenia, Colombia Universidad Antonio Nariño Facultad de Odontología Universidad Antonio Nariño Armenia Colombia

**Keywords:** Periodontitis, oxidative stress, saliva, virus, Aggregatibacter actinomycetemcomitans, Porphyromonas gingivalis, periodontitis, estrés oxidativo, saliva, virus, Aggregatibacter actinomycetemcomitans, Porphyromonas gingivalis

## Abstract

**Introduction:**

Periodontitis affects more than 20% of the Latin American population. Oxidative markers are associated with greater progression of periodontitis; therefore, its role in pathogenesis should be studied.

**Objective:**

To determine the prevalence of the main oral bacteria and viruses associated with periodontitis and estimate the total antioxidant capacity and lipid peroxidation in saliva from patients with periodontitis.

**Materials and methods:**

We conducted systemically a cross-sectional study in 101 healthy subjects, 87 of whom had been diagnosed with periodontitis (P), according to the criteria of the Centers of Disease Control and Prevention and the American Academy of Periodontology, and 14 without periodontal pockets as controls (C). In subgingival samples, major viruses and dental pathogenic bacteria were identified using PCR techniques. The levels of total antioxidant capacity and malon-di-aldehyde (MDA) were determined by spectrophotometry in samples of unstimulated saliva.

**Results:**

The mean of periodontal depth pocket and clinical attachment loss in patients with periodontitis was 5.6 ± 1.7 and 6.1 ± 3.1 mm, respectively. The most prevalent microorganisms were *Aggregatibacter actinomycetemcomitans* (32.5%) and *Porphyromonas gingivalis* (18.6%). The patients from rural areas showed a higher percentage of *A. actinomycetemcomitans* (urban: 17.9% vs. rural: 48.9%, p=0.0018). In patients with periodontitis, the frequency of *EBV, HSV1 & 2,* and *HCMV* genes was 2.3%. Periodontitis patients had higher levels of MDA (P: 2.1 ± 1.5; C: 0.46 ± 0.3 µmol/g protein; p=0.0001) and total antioxidant capacity (P: 0.32 ± 0.2; C: 0.15 ± 0.1 mM; p< 0.0036). Oxidative markers showed no modifications due to the presence of periodontopathic bacteria.

**Conclusions:**

*Aggregatibacter actinomycetemcomitans* was the most prevalent bacteria; its presence did not modify the levels of oxidative markers in the saliva of patients with periodontitis.

## Resumen

**Introducción.:**

La periodontitis afecta a más del 20 % de la población latinoamericana. La presencia de marcadores de estrés oxidativo se asocia con una mayor progresión de periodontitis, por lo que su rol en la patogenia debe estudiarse.

**Objetivo.:**

Determinar la prevalencia de las principales bacterias y virus asociados con la periodontitis y estimar la capacidad antioxidante total y la peroxidación de lípidos en la saliva de los pacientes con periodontitis.

**Materiales y métodos.:**

Se hizo un estudio transversal en 87 sujetos sanos diagnosticados con periodontitis (P) según los criterios de los *Centers of Disease Control and Prevention* y la *American Academy of Periodontology* y 14 sujetos sin enfermedad periodontal como grupo control (C). En las muestras subgingivales se identificaron los principales virus y bacterias mediante técnicas de PCR. Los niveles de capacidad antioxidante total y malon-di-aldehído (MDA) se establecieron mediante espectrofotometría en muestras de saliva no estimulada.

**Resultados.:**

Las medias de profundidad del sondaje y del nivel de inserción clínico periodontal en pacientes con periodontitis fueron 5,6 ± 1,7 y 6,1 ± 3,1 mm, respectivamente. Los microorganismos más prevalentes fueron *Aggregatibacter actinomycetemcomitans* (32,5 %) y *Porphyromonas gingivalis* (18,6 %). Los pacientes de áreas rurales registraron un mayor porcentaje de *A. actinomycetemcomitans* (urbano: 17,9 % *Vs*. rural: 48,9 %; p=0,0018). La frecuencia de los *genes EBV, HSV1 y 2, y HCMV* fue de 2,3 %. En pacientes con periodontitis se evidenciaron mayores niveles de MDA (P: 2,1 ± 1,5; C: 0,46 ± 0,3 µmol/g proteína; p=0,0001) y capacidad antioxidante total (P: 0,32 ± 0,2; C: 0,15 ± 0,1 mM; p<0,0036). La presencia de bacterias periodontales patógenas no modificó los marcadores oxidativos.

**Conclusiones.:**

*Aggregatibacter actinomycetemcomitans* fue el agente patógeno mas prevalente. Su presencia no modificó los niveles de marcadores oxidativos en la saliva de los pacientes con periodontitis.

The periodontal disease is, in fact, a group of infectious and inflammatory progressive conditions that cause the destruction of teeth-supporting tissues such as the periodontal ligament, connective tissue, and alveolar bone ^1^. Periodontitis is the most serious form of periodontal disease affecting 10.8% of the global population ^2^. The highest prevalence (20.4%) and incidence (1,427 cases/100,000 per year) of severe periodontitis are found in Latin America ^3^. The last national survey in oral health in Colombia (ENSAB IV) showed that, in terms of magnitude and impact on general health, periodontitis constitutes the second condition of interest in oral health among the Colombian adult population with a prevalence of 61.8% ^4^.

Periodontitis has a multifactor etiology where infectious and host-related factors such as habits and behaviors, genetics, systemic health, and inflammatory-immune response are involved ^5^. The persistence of microbial pathogens, prolonged inflammatory reaction, and systemic conditions generate oxidative stress ^6^^,^^7^, which is characterized by the increased production of reactive oxygen species ^8^ and the impairment of antioxidant defenses. Several studies have demonstrated the involvement of oxidative stress in the development and progression of periodontitis ^9^^,^^10^. The presence of viruses or virus-bacteria interaction in periodontitis is another possible factor of greater destruction of periodontal tissue ^11^.

In this context, the objective of our study was to determine the biomarkers of oxidative stress in the saliva of periodontitis patients and to estimate the prevalence of the main bacterial and viral dental pathogens. Understanding the local effects on markers of oxidative stress in periodontal disease could be useful for the prevention, diagnosis, and future development of therapeutic adjuvants for this pathology.

## Materials and methods

### Study population

A total of 101 patients between 18 and 65 years of age were recruited consecutively; 87 patients were diagnosed with periodontitis, and 14 with varying degrees of gingival inflammation but no periodontal pockets were selected as controls. Clinical assessments were performed between November, 2017, and November, 2018, in the dental clinic at *Universidad Antonio Nariño* (Bucaramanga) and during an external health campaign in the town of Puerto Wilches in Santander. The patients were invited to participate in the study and recruited if they fulfilled the inclusion criteria and manifested their will to participate by signing an informed consent previously approved by the institutional ethics committee at *Universidad Antonio Nariño*.

The inclusion criteria were: systemically healthy patients over 18 and under 65 years of age, with bleeding upon probing, and periodontal disease. The exclusion criteria were: patients having systemic disorders such as asthma, diabetes mellitus types 1 and 2, HIV, hypo- or hyperthyroidism who had received periodontal therapy in the previous year, had had flu or viral active infection two weeks before or at the moment of collecting the samples or had taken any anti-inflammatory or antibiotic medication in the six months previous to sample collection, and had used antibiotic therapy for routine dental procedures six months prior to the study.

### Periodontitis case definition

We adopted the parameters to define periodontitis of the American Academy of Periodontology and the Centers for Disease Control and Prevention ^12^:

- Gingivitis: without evidence of slight, moderate or advanced periodontitis.
- Slight periodontitis: ≥2 interproximal sites with clinical attachment levels ≥3 mm and ≥2 interproximal sites with probing depth (PD), ≥4 mm (not in the same tooth) or one site with PD≥5 mm.
- Moderate periodontitis: ≥ 2 interproximal sites with clinical attachment levels ≥4 mm (not in the same tooth) or ≥2 interproximal sites with PD≥5 mm (not in the same tooth).
- Advanced periodontitis: ≥2 interproximal sites with clinical attachment levels ≥6 mm (not in the same tooth) and ≥1 interproximal site with PD≥5 mm.

Patients were submitted to a full periodontal examination excluding third molars. Millimetric periodontal probes (PCPUNC156 Hu-Friedy) were used in six different sites considering:

- PD in millimeters from the margin to the bottom of the groove in a range of PD≥4 mm.
- Bleeding upon probing in the percentage of sites with positive bleeding upon probing.
- Clinical attachment levels in millimeters as a result of the distance from the cementoenamel junction to the tip of a periodontal probe during usual periodontal probing.

### Clinical examiners alignment

To validate the periodontal probing examinations performed by two periodontology specialists, we made a calibration to determine both intra- and inter-examiner reliability assessments. The intra-rater reliability was better than inter-rater reliability. Upper intra-rater agreement (exact and within 1 mm) was 0.79 ± 0.14 and lower intra-rater agreement was 0.47 ± 0.17 in 28 tests for all raters. Fleiss’ kappa among three raters was 0.40 with p<0.05.

### Clinical measurements

All patients were examined by a single trained periodontist and probing depth measurements were performed by using a universal North Carolina - 15 periodontal probe (PCPUNC156; Hu - Friedy).

### Saliva samples

Unstimulated whole saliva samples were collected in the morning after a 12-h fasting period or in the afternoon at least 4 h after eating or drinking any food and at least 1 h after dental brushing. In all cases, saliva samples were collected before gingival crevicular fluid samples and after rinsing the mouth with water.

For saliva collection, the subjects were seated comfortably during a 10- min period with restricted conversation and were instructed to allow saliva to accumulate at the bottom of the mouth. Maintaining head tilted slightly forward, saliva samples were collected into a 4-ml polypropylene sterile saliva- collecting vial kept at 4 °C, then transferred to the laboratory and stored at -80 °C until the analyses were performed.

### Selection of sampling sites

The subgingival samples were obtained at the same time of day but after saliva collection. Two or three teeth in different quadrants with the deepest periodontal pocket sites were selected. Before sampling, the individual tooth sites were isolated with cotton rolls and were gently air-dried. The supragingival plaque was carefully removed. Sterilized paper strips #30 and #35 were inserted into the gingival sulcus or periodontal pocket for 20-30 s. In cases of visible contamination with blood, the strips were discarded and a new sample was obtained. Strips from each subject were pooled and placed into labeled tubes containing 300 ml of phosphate buffer solution with a pH of 7.2.

After shaking for 15 min, the strips were removed and the eluates centrifuged for 5 min at 5,800*g* to remove plaque and cellular elements. The samples were then frozen at -80 °C until further biochemical analysis.

### Determination of oxidative stress markers in saliva

*Antioxidant assay analysis.* Before their analysis, all saliva samples were centrifuged (800*g*) for 10 min to separate all cell debris. Total antioxidant capacity of saliva samples was determined by using a total antioxidant assay kit (Ref. CS0790, Sigma-Aldrich Co) based on the conversion of Cu2+ ion to Cu+ by both small molecules and protein antioxidants for its colorimetric detection at ~570 nm.

*Determination of salivary malondialdehyde.* As a measure of lipid peroxidation, levels of salivary MDA were determined by spectrophotometry using a lipid peroxidation assay kit (Ref. MAK085, Sigma-Aldrich).

*Detection and identification of bacterial and viral pathogens.* For DNA isolation, frozen samples from patients were left in 2-ml collection tubes with 0.5 ml sterile distilled water at room temperature for 20 min.

After adjusting the temperature of the samples to room temperature, the sample specimens were pre-clarified by centrifugation to remove debris before DNA extraction using PureLink viral RNA/DNA mini kit™ by Invitrogen and following the protocol recommended by the kit manufacturer for DNA extraction from the tissue samples. DNA concentration, purity, and integrity were verified by using a NanoDrop 2000c™ spectrophotometer (Thermo Scientific).

For bacterial DNA, we performed conventional PCR in a thermocycler ProFlex™ 3 x 32-well PCR System in a final 25-μl volume using OneTaq 2x MasterMix™ (New England Biolabs). For PCR we used specific primers: *A. actinomycetemcomitans,* forward: 5’ AAA CCC ATC TCT GAG TTC TTC TTC 3’ and reverse: 5’ ATG CCA ACT TGA CGT TAA AT 3’*; P. gingivalis,* forward: 5’ AGG CAG CTT GCC ATA CTG CGG 3’ and reverse: 5’ ACT GTT AGC AAC TAC CGA TGT 3’ 13; *Prevotella intermedia,* forward: 5’ TTT GTT GGG GAG TAA AGC GGG 3’ and reverse: 5’ TCA ACA TCT CTG TAT CCT GCG T 3’*;* and *Tannerella forsythia,* forward: 5’ GCG TAT GTA ACC TGC CCG CA 3’ and reverse: 5’TGC TTC AGT GTC AGT TAT ACC T 3’ ^14^.

Amplification was performed under the following conditions: Initial denaturation at 95 °C for 30 s, denaturation at 95 °C for 2 min, and reannealing 60 °C for 1 min; extension temperature at 72 °C for 1 min and 36 cycles. A small fraction (4 µl) of PCR products was mixed properly with 2 µl 6X DNA loading dye (Thermo Scientific), resolved at 90 V/cm on 1.5% agarose gel in 1X TAE buffer for 60 min using a 100 bp DNA ladder and visualized with SYBR Safe DNA Stain Gel™ (Invitrogen) under a UV light in a Spectroline Transilluminator™. *Aggregatibacter actinomycetemcomitans* was identified by the presence of an amplified product in the 505 base pair (pb) band; *P. gingivalis* by an amplified product in the 404 pb band, *P. intermedia* by an amplified product in the 575 pb band, and *T. forsythia* by an amplified product in the 641 pb band.

For the viral genetic detection, a real-time PCR was carried out by using a Bio-Rad CFX96 Touch System™. The amplification was performed by using commercial kits: for Herpes simplex virus (HSV 1 & 2), the herpes simplex virus 1 and 2 DNA polymerase (UL30) gene - genesig standard kit; for Cytomegalovirus (HCMV), the human betaherpes virus 5 - cytomegalovirus, glycoprotein B (gB) gene - genesig standard kit, and for Epstein Barr virus (EBV), the Epstein Barr virus (human herpes virus 4) nonglycosylated membrane protein (BNRF1) - gene genesig advanced kit, which included a positive control template. We used Oasig lyophilised 2X qPCR MasterMixTM. The PCR cycling was performed at 95 °C for 2 min followed by 50 cycles of 95 °C for 10 s and at 60 °C for 60 s. The *R*2 indices were higher than 0.900 in all measurements.

### Statistical analysis

The frequency of periodontopathic microorganisms was recorded as a percentage. The results of oxidative stress markers were presented as means ± standard deviation (SD). Comparisons among groups were evaluated by using the Fischer exact test and the Kruskal-Wallis and Mann-Whitney U tests. Differences were considered statistically significant at p<0.05. Statistical analyses were performed using GraphPad Prism software.

## Results

Table 1 shows the general socio-demographic and clinical characteristics of the study population according to their periodontal status. The average age in patients with chronic periodontitis was higher while the proportion of males and females was similar; 87 patients were screened and diagnosed with chronic periodontitis with 5.6 ± 1.7 mm as the mean of periodontal depth pocket and 6.1 ± 3.1 mm as a mean of clinical attachment loss.

**Table 1:** Socio-demographic and clinical characteristics of the study subjects by periodontal status

Variables	Control	Periodontitis
N	14	87
Age (years)	31±10	45±12
Sex [n (male), %]	6 (42.8)	45 (51.7)
Residence area [n (male), %]		
Urban	9 (64.2)	42 (48.4)
Rural	5 (35.8)	45 (51.6)
Education level [n (male), %]		
Primary school	0	9 (10.3)
Secondary school	4 (28.5)	30 (34.4)
Higher education	10 (71.5)	47 (54.0)
No response	0	1 (1.3)
Marital status [n (male), %]		
Single	8 (57.1)	26 (29.8)
Married/Common law	5 (35.7)	51 (58.6)
Separated/divorced	1 (7.2)	9 (10.3)
No response	0	1 (1.3)
Socioeconomic status [n (male), %]		
Low	6 (42.8)	67 (77.0)
Middle	8 (57.1)	20 (33.0)
Occupation [n (male), %]		
Unemployed	0	13 (14.9)
Working	5 (35.7)	52 (59.7)
Student	9 (64.3)	2 (2.3)
Housewife	0	19 (21.8)
No response	0	1 (1.3)
PPD sampled sites		
(median, media, mm ± S.D.)	2.1, 2.2±0.7	5.0, 5.6±1.7
CAL (median, mm ± S.D.)	1.0, 1.0±0.8	5.0, 6.1±3.1

PPD: Periodontal probing depth; CAL: Clinical attachment level

Table 2 shows the frequency of bacterial and viral periodontopathic microorganisms under evaluation. In all, 56.3% (n=49) of the patients with periodontitis were positive for any of the dental pathogens analyzed; *A. actinomycetemcomitans* and *P. gingivalis* were the most prevalent periodontal pathogens in patients with periodontitis. Codetection of *A. actinomycetemcomitans* and *P. gingivalis* was observed in three patients, but it was not related to the deepest pockets.

**Table 2:** Prevalence of periodontal pathogens according to the periodontal status

Periodontal pathogen	Control n (%)	Periodontitis n (%)	p value*
*P. gingivalis*	0	16 (18.6)	0.117
*A. actinomycetemcomitans*	3 (21.4)	30 (32.5)	0.539
*T. forsythia*	0	3 (3.5)	1.000
*P. intermedia*	0	1 (1.2)	1.000
Epstein-Barr virus	2 (14.2)	2 (2.3)	0.091
Human cytomegalovirus	0	0	-
Herpes virux simplex 1 & 2	1 (7.1)	0	0.138

Fischer exact test

Regarding viruses, the frequency of EBV, HSV 1 and 2, and HCMV in patients with periodontitis was 2.3%; EBV was detected in two patients with chronic periodontitis and EBV and HSV 1 and 2 were identified in two control subjects. The detection of viruses in periodontal pockets was low when there was no co-infection of herpesviruses and bacteria.

**Figure 1:**
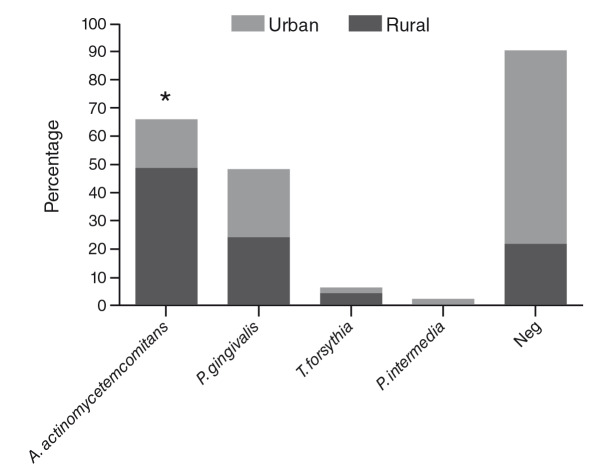
Distribution of periodontal pathogens in patients with periodontitis according to their place of residence

^*^Fisher exact test=0.0027

The most prevalent microorganisms were *A. actinomycetemcomitans* (32.5%) and *P. gingivalis* (18.6%). When we analyzed the distribution of periodontal pathogenic bacteria by residence area, we observed a significantly higher frequency of bacterial pathogens in patients living in rural areas compared with patients living in urban areas (figure 1).

Among patients with periodontitis, 30 (34.5%) showed periodontal pockets with a depth of 4-5 mm and 57 (65.5%) with ≥6 mm. *Porphyromonas gingivalis* had a higher prevalence in patients with periodontal probing depth ≥6 mm; however, no statistically significant differences were observed (table 3).

**Table 3:** Relationship between periodontal probing depth and periodontal pathogens

	4-5 mm n (%)	≥ 6 mm n (%)	p value*
n	30	57	
*P. gingivalis*	3 (10.3)	13 (22.8)	0.245
*A. actinomycetemcomitans*	10 (33.3)	20 (35.1)	0.99

*Fischer exact test

**Figure 2:**
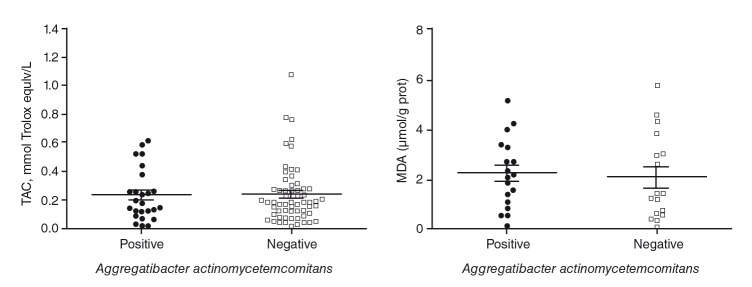
Salivary levels of the total antioxidant capacity (TAC) and salivary malondialdehyde (MDA) in subjects with presence or absence of *A. actinomycetemcomitans*

^*^Statistically significant differences (p<0.05)

Table 4 shows the mean salivary levels of MDA and total antioxidant capacity. The results showed that patients with chronic periodontitis had significantly higher levels of MDA and total antioxidant capacity compared with control subjects. No increase in markers of oxidative stress was observed in the presence of *A. actinomycetemcomitans,* the most prevalent periodontopathic bacteria in this sample of patients (figure 2).

**Table 4:** Analysis of oxidative stress markers according to periodontal status

Marker	Control Mean ± SD	Periodontitis Mean ± SD	p value
TAC mM	0.15 ± 0.1	0.32 ± 0.21	0.0036*
MDA µmol/g prot	0.46 ± 0.3	2.10 ± 1.54	0.0001*

TAC: Total antioxidant capacity; MDA: Salivary malondialdehyde

Comparison of periodontitis vs. control groups by t-test and U Mann-Whitney test

## Discussion

*Porphyromonas gingivalis*, *P. intermedia, A. actinomycetemcomitans*, *T. forsythia,* and *T. denticola* are the most common bacteria associated with the etiology and progression of periodontitis worldwide ^15^^,^^16^. The microbiological profile of the periodontitis they cause and their frequency of distribution differ among geographic regions ^17^^,^^18^ and vary depending on clinical factors such as the depth of the periodontal pockets ^19^.

In Colombia, previous studies have evaluated the microbial profile of the periodontal disease in various regions and in samples collected between 2003 and 2008 and concluded that the most prevalent periodontal pathogens were *P. gingivalis, P. intermedia,* and *T. forsythia*^8^^,^^17^^,^^20^^,^^21^. This study provides further and updated data about the periodontal bacteria and the human viruses associated with periodontitis in Colombian populations.

Our results showed that *A. actinomycetemcomitans* and *P. gingivalis* were the bacteria most often detected in this population versus *P. gingivalis, C. rectus,* and *T. forsythia,* previously reported in patients in Bucaramanga ^8^. Although these periodontal pathogens showed a heterogeneous distribution in this population, *P. gingivalis* remains as one of the main bacteria associated with periodontitis and a reported marker for its progression ^22^. In our study, *A. actinomycetemcomitans* had the highest prevalence and was detected more in the rural population.

This microorganism is one of the bacterial pathogens related to periodontal disease and strongly associated with juvenile periodontitis ^23^, which is considered as the aggressive form of the disease; however, it has also been frequently isolated in chronic periodontitis patients ^24^ with stages 3 and 4, grade B, of the current classification, in those with juvenile gingivitis and even in healthy subjects ^25^. The presence of *A. actinomycetemcomitans* does not always determine a rapid rate of destruction ^26^ and it does not discriminate among periodontitis types or stages ^27^. The pocket depth, a healthy systemic condition in patients or the absence of other disease progression- determining factors may have conditioned our results.

Periodontopathic bacteria and viruses were detected in 48.5% of all subgingival samples, which could be considered low given previous findings in Colombian populations ^8^^,^^17^^,^^20^^,^^28^, and shows the importance of investigating other periodontal pathogens that might be present in the deepest periodontal pockets, as well as the difficulty to obtain adequate subgingival samples. Although many years ago it was demonstrated that “PCR is more accurate than conventional culture methods to identify these periodontal pathogens in subgingival plaque samples” ^29^, the PCR technique, like any other laboratory or diagnostic method, is influenced by internal and external factors such as the integrity of the samples and the DNA concentrations or DNA extraction methods and sample collection techniques. For this reason, we consider that in terms of isolation and detection of periodontal pathogens it is necessary to perform microbiological culture concomitantly with PCR. Culture allows for the detection of unknown pathogens in samples or increases the low concentration of fastidious bacteria DNA obtained due to variations in the amount of subgingival plaque or gingival crevicular fluid recovered from patients.

Other studies in Colombia have shown a higher frequency of viruses in periodontal pockets ^28^^,^^30^ using nested PCR was for virus detection while in our study, we used real-time PCR. PCR methods may have accounted for the differences observed. HCMV was not detected in our samples and the viruses detected showed no coinfection with bacteria. Several studies evaluating associations between periodontopathic bacteria and herpesviruses in patients with juvenile periodontitis have demonstrated that *P. gingivalis* and *A. actinomycetemcomitans* tend to be more frequent in samples showing active infection by herpes viruses, especially HCMV ^31^^,^^32^.

Age is a recognized nonmodifiable risk factor associated with periodontitis ^33^. In our study, patients with periodontitis were older than in the comparison group, which agrees with general descriptions in other populations of patients with periodontal disease in developed and developing countries ^34^. In a previous study in Colombia, differences in terms of the frequency of *P. gingivalis* and *T. forsythia* and the socioeconomic status were found ^20^. Given that most of the population studied here belongs to a low socioeconomic status, our conclusions may be extended to this subset of the general population in Santander, where the distribution patterns of periodontal microorganisms may contribute to establishing focused treatment protocols. However, to reinforce such a conclusion a representative sample should be analyzed.

Regarding salivary markers of oxidative stress, this is the first report in Colombian patients with periodontitis. Whole saliva is an important physiologic fluid useful for the diagnosis and monitoring of many oral and systemic pathological conditions ^35^. Oxidative stress markers in saliva have shown to be a local indicator of the inflammation process, the progression of periodontitis ^36^, and the amount of periodontopathic bacteria in periodontal pockets ^37^.

Our aim was to determine the level of malondialdehyde as a marker of oxidative damage and total antioxidant activity in saliva according to the periodontal status. Our results are similar to those from previous studies ^10^^,^^38^^-^^40^ in the sense that oxidative stress in saliva increased in the periodontitis group as compared with the healthy control group.

The association between salivary oxidative stress markers and the presence of periodontal bacteria had shown a positive correlation before ^41^. We explored this relationship, but we did not observe a positive correlation between total antioxidant capacity and MDA levels in the presence of the most prevalent bacteria: *A. actinomycetemcomitans.* Similarly, and given the low frequency of *P. gingivalis,* it was not possible to correlate its presence with oxidative stress marker levels.

Oxidative stress is caused by the imbalance between the production of reactive oxygen species and the activity of local endogenous antioxidants. Several studies have evaluated the total antioxidant capacity as an indicator of tissue response in periodontitis and have reported its decrease in the saliva of subjects with periodontitis ^42^ while in our study, we observed a higher salivary total antioxidant capacity . Su, *et al*. ^43^ found similar results, which would indicate that higher levels of total antioxidant capacity may be an adaptive response to oxidative stress in some groups of patients. However, unlike other studies, here the saliva samples were taken at two different moments of the day. Several factors can contribute to the variations in the salivary markers, such as the methods of saliva collection, the time of day, the intake of antioxidants, and tooth brushing, which can increase the total antioxidant capacity ^6^^,^^44^. Therefore, additional studies in this population controlling the bacterial load and time of the day for sampling should be conducted.

We can conclude that the patients with periodontitis under study showed differences in the prevalence of *A. actinomycetemcomitans* depending on the place of residence. We also confirmed the increasing levels of oxidative stress and antioxidant protection measured by MDA and total antioxidant capacity in the saliva of patients with periodontitis, regardless of the presence of *A. actinomycetemcomitans*.

Despite the above, further studies are needed in a larger number of patients to evaluate the presence of other pathogenic oral bacteria and to clarify the effect of bacterial presence on salivary oxidative stress, as well as the role of herpesvirusestypes in the region of Santander, Colombia.
